# Co-infection of cytomegalovirus and Epstein–Barr virus-induced pneumonitis following hepatitis B reactivation in an esophageal cancer patient: A case report

**DOI:** 10.1097/MD.0000000000048035

**Published:** 2026-03-27

**Authors:** Quach Thanh Dung, Tran Thanh Tin, Nguyen Huu Thanh, Tran Thi Phuong Thuy, Nguyen Dinh Tung

**Affiliations:** a Department of Oncology, Vinmec Times City International Hospital, Hanoi, Vietnam; b College of Health Sciences, VinUniversity, Hanoi, Vietnam; c Department of Gastroenterology, Vinmec Times City International Hospital, Hanoi, Vietnam; d Department of Infectious Diseases, Vinmec Times City International Hospital, Hanoi, Vietnam.

**Keywords:** cancer, case report, chemoradiotherapy, co-infection, Cytomegalovirus, Epstein–Barr virus, pneumonitis

## Abstract

**Rationale:**

Cytomegalovirus (CMV) and/or Epstein–Barr virus (EBV) infection or reactivation is widely recognized in immunocompromised individuals, particularly those undergoing transplantation or those with acquired immune deficiency syndrome. There are reports of EBV-CMV co-infection or reactivation; however, this co-infection-induced pneumonitis has rarely been seen in patients outside these settings.

**Patient concerns:**

A 64-year-old male with previously treated pulmonary tuberculosis, positive hepatitis B surface antigen and a squamous cell carcinoma of the esophagus underwent 5 cycles of concurrent radiochemotherapy with weekly carboplatin and paclitaxel. Following treatment completion, the patient developed reactivated hepatitis B, requiring antiviral therapy initiation. Six weeks after completing radiochemotherapy, the patient experienced fever and dyspnea.

**Diagnoses:**

The initial chest computed tomography scan revealed honeycomb-like, triangular, mixed alveolar-interstitial opacification in both lungs. Due to a poor clinical response to empiric antibiotics and negative results for both blood and sputum cultures, a bronchoalveolar lavage sample was obtained and revealed high viral loads of EBV and CMV, suggesting EBV-CMV pneumonitis.

**Interventions:**

Treatment with ganciclovir and corticosteroids resulted in significant clinical improvement, with marked resolution of lesions observed on computed tomography scans.

**Outcomes:**

The patient was discharged in stable condition. However, 4 weeks later, while still on corticosteroid taper, the patient developed a recurrence of fever that progressed to respiratory failure, and was subsequently diagnosed with respiratory aspergillosis. The patient passed away after 1 week.

**Lessons:**

This case highlights the importance of suspecting CMV and/or EBV pneumonitis in patients undergoing chemotherapy and no response to empiric antibiotics. Timely detection of causative pathogens is important, as prompt intervention can markedly improve patient outcomes.

## 1. Introduction

Cytomegalovirus (CMV) and Epstein–Barr virus (EBV) are prominent members of the Herpesviridae family, characterized by their double-stranded deoxyribonucleic acid (DNA) genomes. Following primary infection, both viruses possess the ability to establish lifelong latency within host cells. Reactivation from this latent state may occur under specific circumstances, most notably in individuals with compromised immune function.^[1]^ Infection with CMV is a significant cause of severe viral pneumonitis in several immunocompromised patient populations, specifically those who have undergone hematopoietic stem cell transplantation or solid organ transplantation, and individuals with human immunodeficiency virus infection.^[2]^ Diagnosing CMV pneumonitis relies on a combination of diagnostic approaches, which include characteristic radiological patterns observed on imaging, detection of CMV IgM antibodies through serological testing, and the identification of CMV DNA via polymerase chain reaction (PCR).^[3]^ Similarly, EBV pneumonitis is more prevalent in immunocompromised individuals.^[4]^ While the course of EBV infection is typically benign, these vulnerable populations face an elevated risk of pulmonary complications. Approximately 5% to 10% of infectious mononucleosis cases manifest with asymptomatic, mild pneumonitis. However, the occurrence of a severe form of pneumonitis is rare, particularly in immunocompetent individuals.^[5]^ The absence of distinctive clinical manifestations and characteristic imaging features during the early stages of EBV-induced pneumonitis presents significant diagnostic challenges. This often leads to a high propensity for both misdiagnosis and underdiagnosis of the condition.^[6]^

Moreover, pneumonitis induced by CMV and EBV co-infection is rarely reported. There are several cases of this co-infection detected in the blood of patients with leukemia,^[7]^ human immunodeficiency virus,^[8]^ or posttransplantation.^[1]^ A case report has documented concurrent EBV and CMV co-infection in a patient undergoing immunosuppressive treatment with anti-TNFα inhibitors.^[9]^ To the best of our knowledge, this report describes the first case of EBV and CMV co-infection-induced pneumonia in a 64-year-old male patient, occurring after hepatitis B virus reactivation and recent chemoradiotherapy for esophageal squamous cell carcinoma (SCC).

## 2. Case presentation

A 64-year-old male presented with a 2-month history of progressive dysphagia, weight loss and dull epigastric pain. Esophagogastroduodenoscopy revealed a near-complete obstruction in the middle third of the esophagus due to a lesion, through which the endoscope could barely pass. Biopsy confirmed SCC of the esophagus. Subsequent imaging and multidisciplinary tumor board review staged the disease as cT4bN2M0 SCC of the esophagus.

The patient’s medical history included previously treated pulmonary tuberculosis, type 2 diabetes, and chronic obstructive pulmonary disease, both of which were well-controlled on his current medication regimen. Baseline serologic evaluation conducted prior to the initiation of cancer therapy revealed positive hepatitis B surface antigen (HBsAg) and negative hepatitis B e-antigen (HBeAg). Quantitative testing for hepatitis B virus (HBV) DNA demonstrated undetectable viral levels. A shared decision with the clinical oncology team was made to closely monitor liver enzymes throughout the treatment period, rather than initiating prophylactic antiviral therapy. The patient subsequently underwent concurrent chemoradiotherapy, comprising radiotherapy (60 Gy in 30 fractions) combined with weekly carboplatin (AUC2) and paclitaxel (50 mg/m^2^) for a duration of 5 weeks. The patient successfully completed the full course of treatment without any reported complications.

At a routine follow-up 4 weeks after completing chemoradiotherapy, the patient reported new-onset fatigue and unexplained fever. Initial laboratory testing revealed elevated transaminases, with aspartate aminotransferase (AST) and alanine aminotransferase (ALT) levels elevated to 608.7 U/L and 554.4 U/L, respectively. Quantitative HBV DNA testing confirmed a viral load of 2.3 × 10^7^ copies/mL, leading to a diagnosis of reactivated hepatitis B virus (HBV). Antiviral therapy with tenofovir alafenamide (TAF) 25mg/day was promptly initiated.

**Figure 1.**
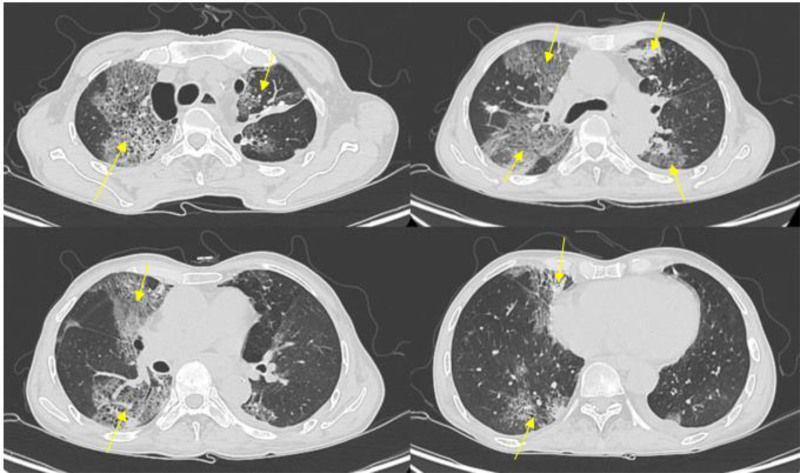
Initial chest CT scan: honeycomb-like triangular mixed alveolar-interstitial opacification in both lungs (thin yellow arrow).

**Figure 2.**
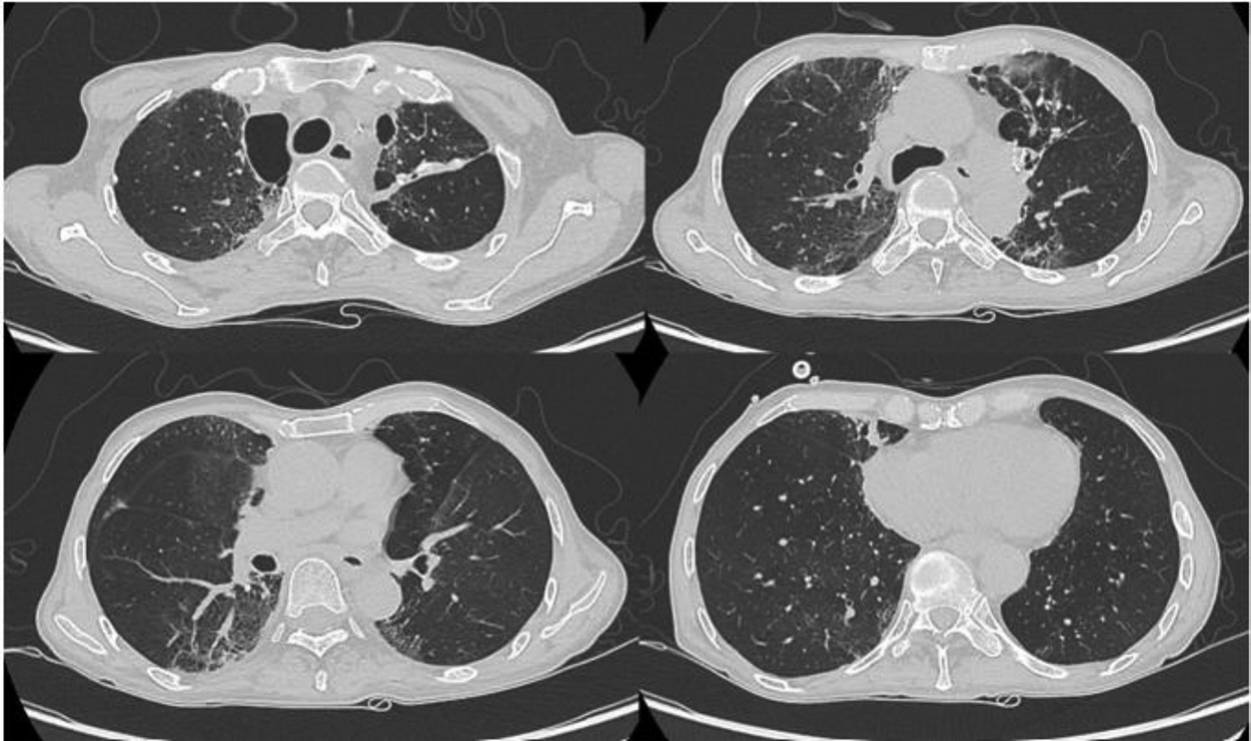
The chest CT scan after 1 week of intravenous ganciclovir and glucocorticoid therapy: Notable improvements in the same locations for comparison with the initial chest CT scan.

At the 6-week follow-up following completion of chemoradiotherapy, the patient reported ongoing fevers up to 38.6°C accompanied by worsening shortness of breath. On physical examination, bilateral coarse crackles were noted on lung auscultation. A clinical diagnosis of pneumonia was established. Initial blood tests revealed in Table 1. The initial chest CT scan revealed the new honeycomb-like triangular mixed alveolar-interstitial opacifications characterized by consolidation, ground-glass opacity, septal thickening, and bronchiectasis in both lungs (Fig. 1). The patient was empirically started on intravenous piperacillin-tazobactam and oral azithromycin due to a high risk of multidrug resistance. Due to a poor clinical response after 48 hours, the treatment was escalated to intravenous meropenem, with the addition of intravenous fluconazole and vancomycin. Despite these measures, blood and sputum cultures failed to identify any causative pathogens, and the respiratory viral PCR (including The BIOFIRE® FILMARRAY® Pneumonia (PN) Panel Menu) was negative. Quantitative CMV DNA PCR using a blood sample showed undetectable viral DNA. The patient still had 2 to 3 episodes of fever per day and required increased oxygen therapy. Other specific respiratory pathogens and causes were suspected, including CMV, EBV, tuberculosis, radiation, and inflammatory diseases. To further investigate, bronchoalveolar lavage was performed. PCR testing of the bronchoalveolar lavage sample confirmed the presence of CMV (4.31 × 10^4^ copies/mL) and EBV DNA (6.55 × 10^4^ copies/mL), establishing the diagnosis of EBV-CMV co-infection. No other causative pathogens were identified through BLA culture or molecular testing, including Tuberculosis, bacteria, and fungi (Table 2). The patient was initiated on intravenous ganciclovir at a dose of 500 mg/day combined with methylprednisolone 40 mg/day, which resulted in notable clinical improvement. After 1 week of treatment, a follow-up chest CT scan demonstrated significant resolution of pulmonary lesions (Fig. 2). Consequently, the patient was discharged on oral valganciclovir 1800 mg/day and methylprednisolone 32 mg/day.

**Table 1:** Laboratory results on admission and on the day of CMV–EBV pneumonitis diagnosis (day 10).

Blood works	Admission day	Day 10	Reference range
RBC	3.22 T/L	3.2 T/L	4.2–5.4
HGB	106 g/L	100 g/L	120–150
WBC	5.4 G/L	3.5 G/L	4–10
Neutrophil count	3.70 G/L	2.47 G/L	1.7–7.5
Lymphocyte count	1.01 G/L	0.59 G/L	0.9–7.5
PLT	165 G/L	216 G/L	150–450
AST	411.5 U/L	259.2 U/L	<50
ALT	366.5 U/L	193.1 U/L	<50
Alkaline phosphatase	234 U/L	–	30–120
INR	1.24	–	–
Bilirubin – total	9.31 μmol/L	–	0–21
Bilirubin – direct	5.06 μmol/L	–	0–5
Bilirubin – indirect	4.30 μmol/L	–	0–16
C-reactive protein	50 mg/L		<3
Pro-calcitonin	–	0.225 ng/mL	<0.05

ALT = alanine aminotransferase, AST = aspartate aminotransferase, CMV = cytomegalovirus, EBV = Epstein-Barr virus, HGB = hemoglobin concentration, INR = international normalized ratio, PLT = platelet count, RBC = red blood cell count, WBC = white blood cell count.

**Table 2:** Pathological investigations during the first admission.

Test	Results	Remarks
Sputum		
Sputum culture	Negative	No growth detected
Respiratory pathogens panel (PCR)*	Negative	No respiratory pathogen identified
Xpert MTB/RIF assay	Negative	No evidence of tuberculosis
Blood		
Quantitative CMV/EBV DNA PCR	Undetected	No evidence of systemic CMV/EBV infection
Serum galactomannan	Negative	No evidence of systemic Aspergillosis
Blood culture	Negative	No bacteria growth detected
BAL		
CMV DNA PCR	4.31 × 10^4^ copies/mL	CMV confirmed in lower respiratory tract
EBV DNA PCR	6.55 × 10^4^ copies/mL	EBV confirmed in lower respiratory tract
Ziehl–Neelsen stain	Negative	Negative
MGIT culture	Negative	No evidence of tuberculosis
Xpert MTB/RIF assay	Negative	No evidence of tuberculosis
Bacterial culture	Negative	No respiratory bacteria identified in lower respiratory tract
Fungi culture	Negative	No respiratory fungi identified in lower respiratory tract

BAL = bronchoalveolar lavage, CMV = cytomegalovirus, DNA = deoxyribonucleic acid, EBV = Epstein–Barr virus, MGIT = Mycobacteria growth indicator tube, PCR = polymerase chain reaction, Xpert MTB/RIF assay = rapid molecular test for mycobacterium tuberculosis detection and rifampicin resistance.

* Including The BIOFIRE® FILMARRAY® Pneumonia (PN) Panel Menu: 33 targets (18 bacteria, 8 viruses, 7 antimicrobial resistance genes).

**Figure 3.**
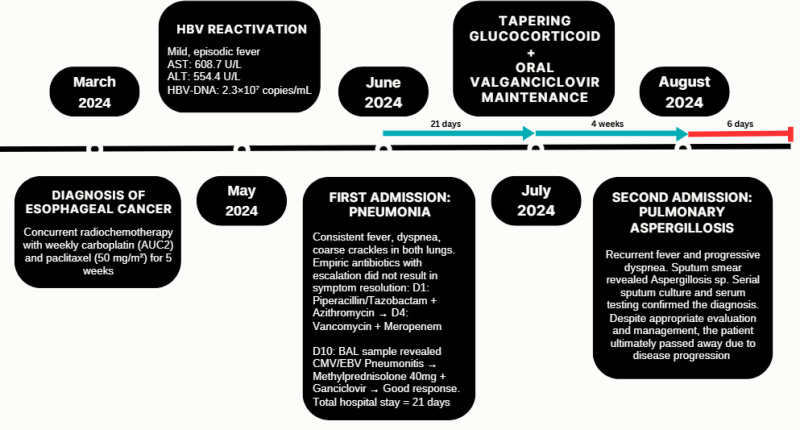
Timeline of the patient’s clinical course, follow-up, and outcome.

However, 4 weeks later, while tapering methylprednisolone at 16mg/day, the patient developed a recurrence of fever and progressive dyspnea. The patient subsequently developed respiratory failure, necessitating admission to the intensive care unit. Bronchoalveolar lavage testing confirmed a diagnosis of respiratory aspergillosis. Due to the severity of the disease, the patient passed away 1 week later. The timeline of the patient’s clinical course is shown in Figure 3.

## 3. Discussion

To the best of our knowledge, our case of EBV and CMV-induced pneumonitis in a patient with esophageal cancer undergoing standard chemoradiotherapy is the first case reported. This case highlights that the prophylaxis of HBV reactivation should be considered before chemotherapy based on the risk of HBV reactivation. Clinicians should suspect CMV and/or EBV pneumonitis in patients receiving cancer treatment who fail to respond to empiric antibiotics. The early recognition of EBV-CMV pneumonitis leads to prompt antiviral therapy, which will reduce the severity and mortality rate. Corticosteroids may be used in selected patients with close follow-up for the prevention of opportunistic infections.

CMV pneumonia in the setting of non-transplantation patients is a rarity. The clinical manifestations are largely nonspecific and can overlap with other infectious etiologies, making a definitive diagnosis challenging. Regarding diagnosis, quantitative real-time PCR (qRT-PCR) can be employed to quantify viral loads in both blood and bronchoalveolar lavage (BAL) fluid. BAL CMV-PCR is widely regarded as the most accepted approach for viral isolation within the lungs due to its high sensitivity. While less commonly performed, lung biopsy histopathology, demonstrating the presence of characteristic CMV inclusions, remains the gold standard for diagnosing pulmonary CMV infections.^[2,10]^

Similarly, EBV-associated pneumonitis is rare but potentially life-threatening. EBV in adults mainly presents as a latent infection. The early presentation of EBV pneumonitis typically involves nonspecific symptoms resembling other viral pneumonia. Furthermore, definitive radiological features are frequently absent, with most chest CT scans revealing only ground-glass opacities, which contribute to misdiagnosis and delayed therapeutic intervention.^[4]^ The diagnosis of EBV pneumonitis hinges on suggestive clinical symptoms coupled with positive serological testing or confirmed quantitative EBV DNA measurements, while concurrently excluding other potential etiologies of pneumonia. While EBV-encoded small RNA (EBER) in situ hybridization is considered the gold standard for detecting EBV in tissue samples, its clinical utility is limited by its invasive nature.^[11]^

Our case is unique for several reasons. Firstly, isolated EBV or CMV-induced pneumonitis is rare; consequently, CMV and EBV co-infection-induced pneumonitis are exceedingly unusual, particularly in patients with esophageal cancer undergoing radiochemotherapy. Other reported cases of EBV and CMV co-viremia have primarily involved immunocompetent patients or those receiving adalimumab, with subsequent development of hemophagocytic lymphohistiocytosis and without evidence of pneumonia.^[9,12]^ Secondly, reactivation of HBV may be the initial indicator for an impaired immune system, followed by subsequent EBV and CMV-induced pneumonitis and respiratory aspergillosis. Furthermore, after reviewing our case, the prophylaxis of HBV reactivation should be considered before chemotherapy. It is recommended that antiviral therapy be administered concurrently or prior to initiating immunosuppressive therapy to patients who are at moderate to very high risk of HBV reactivation.^[13]^ Thirdly, the early diagnosis of CMV–EBV-induced pneumonitis was quite challenging. Due to the nonspecific symptoms and bilateral infiltration on CT scan, we had to differentiate them from multiple diseases. The most likely diagnosis was pneumonia caused by bacteria and fungi. Moreover, drug-induced and radiation-induced pneumonitis should be considered as a differential diagnosis due to the given timing of the last dose of chemotherapy (carboplatin and paclitaxel) and radiation therapy. The similar symptoms and chest CT scan made it difficult to rule them out completely because they were a diagnosis of exclusion. Although the patient did not undergo lung tissue biopsy and lacked pathologic information, these clinical manifestations and good response with ganciclovir were consistent with EBV and CMV-induced pneumonitis. This diagnostic challenge mirrors the findings of Huibin Liao et al, who reported a case of EBV pneumonitis in a breast cancer patient undergoing neoadjuvant chemotherapy. In their case, establishing a definitive diagnosis was hindered by negative microscopic findings in stained sputum and bronchoalveolar lavage fluid (BALF) smears. The diagnosis of EBV pneumonitis was ultimately suspected and confirmed only after PCR analysis of the BALF revealed a high viral load.^[4]^

Although no specific antiviral agents are approved for EBV and CMV co-infection-induced pneumonitis, available literature suggests that ganciclovir, acyclovir, and corticosteroid therapy have demonstrated favorable clinical outcomes in reported cases.^[1,4,9]^ The prognosis of CMV disease alone is generally good if the pathogen is correctly addressed. Early initiation of therapy significantly improves outcomes, as demonstrated by Kanika et al, who reported an 89% recovery rate in treated cases of CMV-induced pneumonitis.^[2]^ Our case also showed that the patient improved quickly after 1 week of ganciclovir and corticosteroids. This evidence may help further studies to investigate the appropriate treatment for EBV and CMV co-infection-induced pneumonitis. However, the role and duration of corticosteroid use were unclear in the treatment for EBV and CMV co-infection-induced pneumonitis. There is a case report showing that EBV-induced pneumonitis was successfully treated with corticosteroids alone, especially in the delayed diagnosis.^[14]^ Similarly, corticosteroids continue to play a role in the management of CMV-induced pneumonitis^[15]^ and in a case with nonspecific presentations that overlap with immune-mediated pneumonitis.^[16]^ However, corticosteroids may exert immunosuppressive effects, potentially increasing the risk of CMV reactivation and subsequent infection in susceptible individuals. Our patient used corticosteroids because of the delayed diagnosis of EBV, severely impaired lung function, and overlapped drug and radiation-induced pneumonitis. However, the patient subsequently became infected with aspergillosis. Therefore, corticosteroids may be used in selected patients with close follow-up, particularly those with an immunocompromised state and viral infections. Further studies are needed on the optimal treatment approach for CMV–EBV co-infection in pneumonia, the role of corticosteroids in pneumonia in this context, as well as strategies to minimize secondary infectious complications.

## 4. Conclusions

This report highlights an unusual case of EBV and CMV co-infection causing pneumonia in a patient with esophageal cancer undergoing standard chemoradiotherapy. This case also underscores the importance of considering HBV reactivation prophylaxis prior to the initiation of chemotherapy, particularly in patients at risk. Clinicians should maintain a high index of suspicion for EBV and CMV pneumonitis in immunocompromised patients, especially those who fail to respond to empiric antibiotic therapy. Early identification of EBV-CMV pneumonitis allows for the timely initiation of antiviral treatment. The use of corticosteroids may be considered in selected cases, with careful monitoring for potential opportunistic infections.

## Author contributions

**Conceptualization:** Quach Thanh Dung, Tran Thanh Tin, Nguyen Huu Thanh, Tran Thi Phuong Thuy.

**Data curation:** Tran Thanh Tin, Nguyen Huu Thanh, Nguyen Dinh Tung.

**Investigation:** Tran Thanh Tin, Nguyen Huu Thanh, Nguyen Dinh Tung.

**Methodology:** Quach Thanh Dung, Tran Thanh Tin, Nguyen Huu Thanh, Tran Thi Phuong Thuy, Nguyen Dinh Tung.

**Project administration:** Quach Thanh Dung, Nguyen Huu Thanh, Tran Thi Phuong Thuy.

**Resources:** Quach Thanh Dung.

**Supervision:** Quach Thanh Dung, Nguyen Huu Thanh, Tran Thi Phuong Thuy.

**Validation:** Quach Thanh Dung, Nguyen Huu Thanh, Tran Thi Phuong Thuy.

**Visualization:** Quach Thanh Dung, Nguyen Huu Thanh, Tran Thi Phuong Thuy.

**Writing – original draft:** Quach Thanh Dung, Tran Thanh Tin, Nguyen Huu Thanh, Tran Thi Phuong Thuy, Nguyen Dinh Tung.

**Writing – review & editing:** Quach Thanh Dung, Tran Thanh Tin, Nguyen Huu Thanh, Tran Thi Phuong Thuy, Nguyen Dinh Tung.
